# Efficacy of Human Exposures of Gepotidacin (GSK2140944) against *Escherichia coli* in a Rat Pyelonephritis Model

**DOI:** 10.1128/AAC.00086-19

**Published:** 2019-06-24

**Authors:** Jennifer L. Hoover, Christine M. Singley, Philippa Elefante, Stephen Rittenhouse

**Affiliations:** a GlaxoSmithKline, Collegeville, Pennsylvania, USA

**Keywords:** gepotidacin, pyelonephritis, urinary tract infection

## Abstract

Gepotidacin is a first-in-class triazaacenaphthylene antibacterial that inhibits bacterial type II topoisomerases and has *in vitro* activity against a range of bacterial pathogens, including Escherichia coli. Urinary tract infections often progress to pyelonephritis and are a worldwide problem due to the prevalence of multidrug-resistant E. coli strains. This study evaluated the *in vivo* efficacy of gepotidacin against four strains of multidrug-resistant E. coli in a rat pyelonephritis model. Infected rats received controlled intravenous infusions of gepotidacin every 12 h for 4 days that recreated human systemic exposures from oral gepotidacin (800 or 1,500 mg twice daily for 4 days). Liquid chromatography-tandem mass spectrometry analysis of blood samples and kidney homogenates showed that gepotidacin levels were 6- to 7-fold higher in kidneys than in blood. Across experiments with 4-day gepotidacin treatments, bacterial CFU in kidneys were reduced by 2.9 to 4.9 log_10_ compared to pretreatment levels, and bladder CFU were reduced to the lower limit of detection (1.2 log_10_). The efficacies of 800- and 1,500-mg gepotidacin exposures were statistically similar. A time-course experiment indicated that a period of more than 24 h of gepotidacin treatment was required for efficacy and that 4 days were needed for maximal response. Overall, these results demonstrate that the recreated human exposures of gepotidacin studied were effective in an animal model of pyelonephritis caused by multidrug-resistant E. coli and that further evaluation for clinical use is warranted.

## Abstract (precis)

Gepotidacin is a first-in-class triazaacenaphthylene antibacterial that inhibits bacterial type II topoisomerases and has *in vitro* activity against a range of bacterial pathogens, including Escherichia coli. Urinary tract infections often progress to pyelonephritis and are a worldwide problem due to the prevalence of multidrug-resistant E. coli strains.

## INTRODUCTION

Urinary tract infections (UTIs) are extremely common, affecting ∼150 million people per year worldwide, and represent the most common type of bacterial infection in women and in older populations (1). UTIs are a significant public health problem due to their prevalence and high rates of treatment resistance and recurrence.

Gepotidacin (GSK2140944), a first-in-class, novel triazaacenaphthylene antibacterial agent, is being explored as a novel treatment option for UTI due to its *in vitro* activity against Escherichia coli, the most common UTI pathogen (2, 3). Gepotidacin inhibits bacterial type II topoisomerases, thereby selectively blocking bacterial DNA replication (4). It inhibits bacterial DNA gyrase and topoisomerase IV via a unique mechanism of action that is not shared by any other available antibiotics (4); thus, it retains activity against drug-resistant bacterial strains associated with a range of conventional and biothreat infections, including cephalosporin-, fluoroquinolone-, and carbapenem-resistant isolates of E. coli (2, 3). Oral and intravenous (i.v.) formulations of gepotidacin have been developed and evaluated for pharmacokinetics (PK), metabolism, and disposition in healthy human subjects (5). In addition, phase 2 studies have demonstrated efficacy, safety, and tolerability of gepotidacin in patients with acute bacterial skin and skin structure infection (6) and those with uncomplicated gonorrhea (7).

We investigated the potential clinical utility of gepotidacin as a treatment for UTIs caused by multidrug-resistant (MDR) E. coli by evaluating the efficacy of recreated human exposures of the drug in a rat model of pyelonephritis. Recreated exposure profiles targeting the concentration-time curves measured in healthy humans following twice-daily (BID) administration of 800- or 1,500-mg oral doses (8) were tested against four MDR strains of E. coli. The effect of treatment duration on efficacy and the concentration of gepotidacin in rat kidney homogenates was also determined with dosing targeting the 1,500-mg human exposure profile.

## RESULTS

### Recreation of human oral dose exposures of gepotidacin in infected rats using controlled i.v. infusion.

Four MDR E. coli isolates were used in this study to induce pyelonephritis in rats by direct injection of bacterial suspensions into the kidneys. These four isolates were previously determined to have MICs of 2 to 4 μg/ml for gepotidacin and were resistant to levofloxacin (MIC = 16 to 32 μg/ml) *in vitro* (Table 1).

**TABLE 1:** MICs of gepotidacin and levofloxacin

E. coli isolate	Genotype^*a*^	MIC (μg/ml)	
		Gepotidacin	Levofloxacin
NCTC13441	ST-131	4	16
5649	NDM-1	2	32
IR5	NDM-1	4	32
ALL	NDM-1	4	32

a NDM-1, New Delhi metallo-β-lactamase 1; ST-131, sequence type 131.

**FIG 1:**
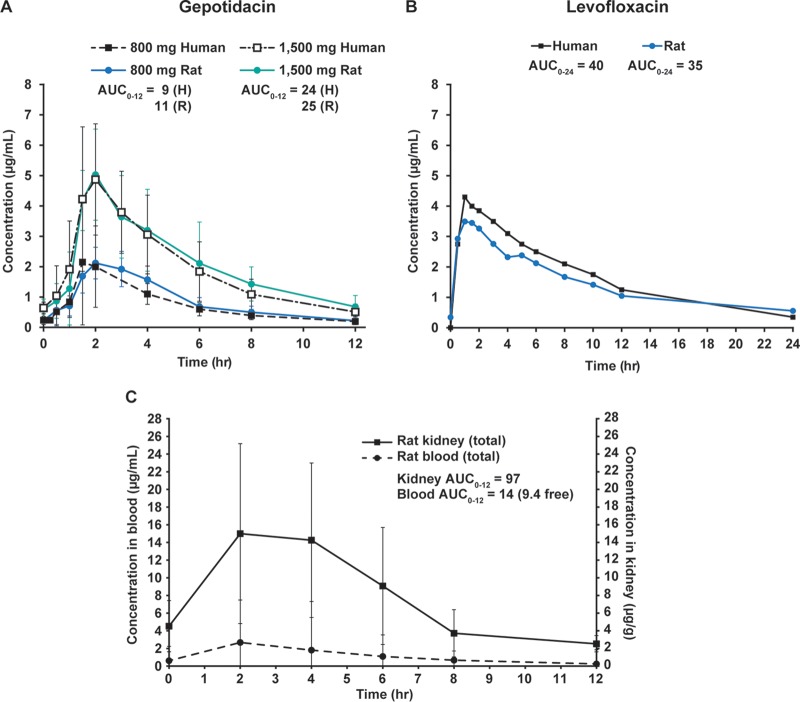
Concentration-time curves illustrating systemic exposure to gepotidacin and levofloxacin, and gepotidacin concentrations in blood and kidney homogenates from infected rats following controlled i.v. drug infusion. E. coli-infected rats were treated with gepotidacin q12h targeting 800- or 1,500-mg twice-daily human oral dose exposures (A) or with levofloxacin q24h targeting human exposure with a once-daily 500-mg oral dose (B). Blood samples were collected for measurement of drug levels in plasma by HPLC-MS/MS at the indicated times after the third dose of gepotidacin or after the second dose of levofloxacin. Previously established human concentration-time curves are shown for reference (black lines and symbols). Mean concentrations ± the SD for total drug are shown (*n* = 5 to 6 rats/time point) for all E. coli experiments pooled. (C) Blood samples and kidneys were collected for measurement of drug levels by HPLC-MS/MS at the indicated times after the third dose of gepotidacin in a separate (nonefficacy) experiment. The mean total drug concentrations in blood (μg/ml) and in kidney (μg/g) ± the SD are shown (*n* = 3 rats/time point). AUC_0–24_, area under the concentration-time curve from time zero to 24 h; AUC_0–12_, area under the concentration-time curve from time zero to 12 h; H, human; hr, hours; R, rat.

Systemic exposures for gepotidacin were evaluated on the second day of dosing during all experiments. Gepotidacin exposure profiles from each individual experiment are shown in Fig. S1A, S2A, S3A, and S4A in the supplemental material; data were pooled, and the overall mean profile was similar to the target human exposures for gepotidacin (Fig. 1A) and for levofloxacin (Fig. 1B). Confidence intervals (95% upper and lower bounds) were also calculated for gepotidacin and are shown in Fig. S5.

In addition, gepotidacin concentrations were measured in the kidneys of rats infected with E. coli (NCTC13441) when gepotidacin was dosed to the target 1,500-mg human exposure (Fig. 1C). Total drug concentration in the kidney was 5.6-fold higher (based on the maximum concentration [*C*_max_]) and 6.8-fold higher (based on area under the concentration-time curve from time zero to 12 h [AUC_0–12_]) compared to the blood concentration. Although the kidney exposures appear to be quite variable, this is likely because the samples were, by necessity, terminal; therefore, each data point was provided by a different rat. The overall AUCs per kidney calculated from composite exposure profiles were similar.

### Efficacy of human exposure levels of gepotidacin against MDR *E. coli* isolates in the rat pyelonephritis model.

**FIG 2:**
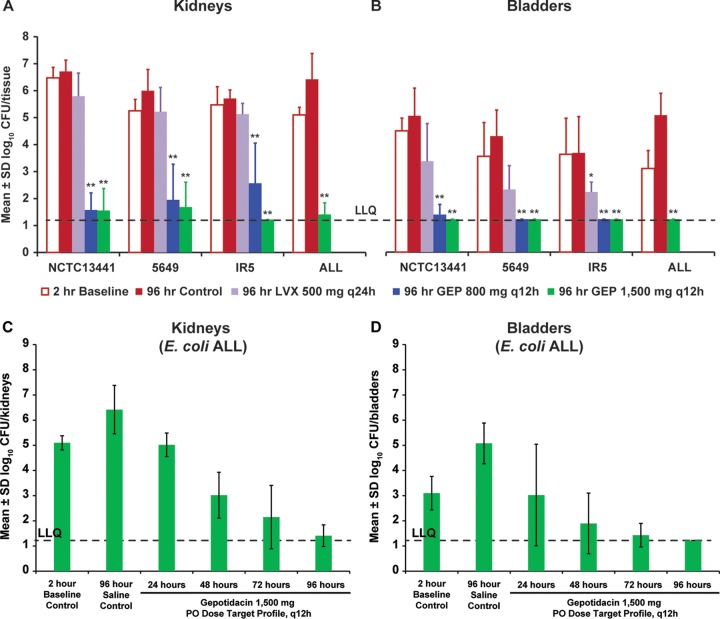
Bacterial burden after 4 days of treatment and time course of gepotidacin efficacy in rat kidneys and bladders. Rats with pyelonephritis induced by one of four different MDR E. coli isolates were treated for 4 days with saline (“control”), levofloxacin, or gepotidacin. All CFU assays were performed using kidneys (A) and bladders (B) collected from animals euthanized at 2 h postinfection (prior to treatment initiation, “baseline”) or at 96 h (at end of final infusion). Mean ± the SD log_10_ CFU/organ (each kidney pair pooled per animal) is shown (*n* = 5 to 6 rats per time point). Neither levofloxacin nor the 800-mg exposure profile of gepotidacin were tested against E. coli ALL. Asterisks (*, *P* < 0.05; **, *P* < 0.01) represent a statistically significant reduction versus 2-h baseline controls. Rats with pyelonephritis induced by E. coli ALL were treated with i.v. saline (“control”) or gepotidacin as a recreated exposure profile targeting human 1,500-mg oral (PO) q12h dosing. All CFU assays were performed using kidneys (each pair pooled per animal) (C) and bladders (D) collected from animals euthanized at 2 h postinfection (prior to treatment initiation, “baseline”) or at 24, 48, 72, or 96 h. *n* = 5 to 6 rats/time point. hr, hours; GEP, gepotidacin; LLQ, lower limit of quantification; LVX, levofloxacin.

The efficacies of 800- or 1,500-mg oral dosing profiles of gepotidacin against pyelonephritis are shown in Fig. 2A and B. Across all studies, the mean bacterial burden in 2-h baseline controls ranged from 5.1 to 6.5 log_10_ CFU/kidneys and 3.1 to 4.5 log_10_ CFU/bladders. After 4 days of saline treatment, bacterial levels remained similar or increased (mean CFU ranges, 5.7 to 6.7 log_10_ CFU/kidneys and 3.7 to 5.1 log_10_ CFU/bladders). Overall, for both gepotidacin exposures (800- and 1,500-mg recreated oral dose) and all four E. coli isolates, gepotidacin treatment led to profound (2.9- to 4.9-log_10_) reductions in kidney CFU compared to baseline (nontreated) controls (Fig. 2A), and reduced bladder CFU to near or below the lower limit of quantification (LLQ; Fig. 2B). In contrast, but as expected given the levofloxacin resistance of the isolate, treatment with levofloxacin every 24 h (q24h) at the recreated human 500-mg once-daily oral dose did not reduce bacterial burden in the kidneys (Fig. 2A) and had a much less pronounced effect compared to gepotidacin in the bladder (Fig. 2B). Detailed efficacy data for each bacterial isolate are shown in Fig. S1B, S2B, S3B, and S4B.

The 1,500- and 800-mg gepotidacin target exposures were similarly effective in reducing bladder and kidney CFU in this model, resulting in CFU/organ counts that were not statistically different from one another by Student *t* test (*P* > 0.05). The log_10_ CFU/kidneys at the 96-h time point for 800-mg versus 1,500-mg gepotidacin exposures were 1.6 ± 0.6 versus 1.6 ± 0.8 for E. coli isolate NCTC13441, 2.0 ± 1.3 versus 1.7 ± 0.9 for E. coli isolate 5649, and 2.6 ± 1.5 versus ≤1.2 for E. coli isolate IR5. Although not statistically significant, there was an apparent correlation between higher AUC values and both greater reductions in kidney CFU and less variability between animals in individual experiments, particularly in animals infected with E. coli isolate IR5.

### Effect of treatment duration on gepotidacin efficacy against MDR *E. coli* in the rat pyelonephritis model.

The effect of the gepotidacin 1,500-mg oral exposure profile on a q12h schedule was assessed at 24, 48, 72, or 96 h after the start of treatment against E. coli ALL. Overall, there was a positive correlation between the duration of treatment and antibacterial efficacy in both kidneys (Fig. 2C) and bladders (Fig. 2D). Compared with baseline levels, bacterial counts were reduced by 0.1, 2.1, 2.9, and 3.7 log_10_ CFU/kidneys and by 0.1, 1.2, 1.7, and ≥1.9 log_10_ CFU/bladder after 24, 48, 72, or 96 h of gepotidacin treatment, respectively. Thus, the drug had little effect on the bacterial burden within the first 24 h of dosing, but each subsequent day of dosing resulted in additional CFU reduction, with maximal effect reached in both kidneys and bladders after 4 days of treatment.

## DISCUSSION

The increasing prevalence of MDR strains of E. coli has far exceeded the pace of discovery and development of new antimicrobial agents that are active against common drug-resistant isolates. This has led to a growing worldwide crisis in which common infections such as UTIs are becoming less treatable, resulting in increasing morbidity, mortality, and economic burden. Gepotidacin is a promising candidate to meet the need for new UTI treatments due to its unique mechanism of action, its ability to be administered in both i.v. and oral formulations, its *in vitro* activity against a broad spectrum of bacterial pathogens (including MDR strains of E. coli), and its low spontaneous frequency of resistance *in vitro* (for most organisms, including E. coli) (2, 4, 5, 9). Moreover, gepotidacin has been studied for safety and pharmacokinetics in human volunteers (5) and for efficacy in acute bacterial skin and skin structure infections, as well as uncomplicated urogenital gonorrhea (6–8).

The objective of the present study was to evaluate the efficacy of gepotidacin using an animal model physiologically related to human UTIs. In the selected model, pyelonephritis is induced in rats by direct injection of bacterial suspension into the kidneys (10). This model better represents the physiology of UTI compared to other commonly used experimental systems, such as single-compartment *in vitro* models and the neutropenic mouse thigh infection. In addition, direct injection of kidneys reduces the variability of infection often observed in transurethral models of UTI (11–13). We also delivered gepotidacin by controlled i.v. infusion to recreate systemic exposures measured in a previous phase 1 study after oral administration of gepotidacin to healthy volunteers (8). The advantages of incorporating human PK data into animal studies during pharmaceutical development was demonstrated in our previous work (14) and can provide data useful for guiding the selection of dosing regimens most likely to be effective in clinical studies, thus reducing the risk associated with the clinical development of new antibacterial agents (14, 15). Together, these aspects of our study design suggest that the obtained data are clinically relevant in that they demonstrate the effects of drug levels achievable in humans against infection involving a clinically relevant site.

Comparison of whole blood concentrations of gepotidacin in rats dosed by controlled i.v. infusion q12h in this study versus those observed in humans after BID oral dosing with 800 or 1,500 mg (8) demonstrated that human exposure profiles were successfully recreated in rats. This was also achieved for levofloxacin i.v. infusion q24h targeting the human exposure profile for a once-daily oral dose of 500 mg. For both gepotidacin (data on file) and levofloxacin (16, 17), concentrations in whole blood and plasma have been shown to be similar; therefore, additional adjustments for blood-plasma partitioning are not necessary. Concentrations of gepotidacin in kidney homogenates of rats dosed to the 1,500-mg oral human exposure target were ∼6-fold higher than in whole blood based on total drug *C*_max_ and ∼7-fold higher based on total drug AUC_0–12_. These results show that gepotidacin is present and active in the kidney, a clinically important tissue in many UTIs. Although the presence and possible accumulation of gepotidacin in the kidney are important, data from analysis of tissue homogenates should be considered with caution (12, 18, 19).

Gepotidacin treatment resulted in a profound reduction in bacterial CFU in kidneys collected from infected rats. In addition, CFU in the bladders of gepotidacin-treated rats were reduced to near or below the limit of detection. This was consistently observed across experiments, for both the 800- and 1,500-mg target exposures and for all four E. coli isolates tested. In a time course experiment in which kidney and bladder CFU were measured daily during q12h treatment targeting 1,500-mg oral human exposure, CFU were not reduced below baseline levels until the 48-h time point but showed increasing reductions at each subsequent time point, with the maximal effect observed at 96 h. The apparent delay in bacterial killing from the start of treatment in this animal model is surprising, given the bactericidal mechanism of action for gepotidacin (3) and the presence of an intact immune system in the rats. A similar delay in effect has been observed with other novel, bactericidal compounds in this model (data not shown); this may suggest it is a model-related event (e.g., associated with the direct injection of a large bacterial bolus into the kidney, which may alter the structure of the organ and is not the natural course of disease). The translation of this observation to clinical outcome is not currently known, and further exploration of this rat pyelonephritis model using well-established antibacterial agents would be useful.

Overall, the results showed that the efficacy of the recreated 800- and 1,500-mg gepotidacin exposure profiles were similar and not statistically different in the rat pyelonephritis model. Pooled mean daily blood AUC values achieved in rats were 21 and 51 μg ⋅ h/ml (free drug, 14 and 34 μg ⋅ h/ml), respectively, for these tested profiles. In general, maximum efficacy (bacteria reduced close to or below the limit of detection) was achieved at the higher dose, and there was more variability at the lower dose. Of the two doses studied, 800 mg represents the minimum efficacious dose; therefore, a daily free-drug AUC of approximately 14 μg ⋅ h/ml (95% confidence interval, 12 to 16 μg ⋅ h/ml) could be considered when setting pharmacokinetic/pharmacodynamic (PK/PD) targets. The exposure profiles for only two selected candidate dosing regimens were evaluated in these studies, and additional work is planned to characterize the full exposure response range in experimental models more amenable to that goal. The integration of these data sets will provide complementary information and support a robust dose rationale informed by multiple different types of experiments, including investigation of efficacy and prevention of resistance. Data have already been generated which demonstrate that the ratio of the free AUC to the MIC is the PK/PD index most closely associated with gepotidacin efficacy against E. coli in a one-compartment *in vitro* infection system (9) and in a mouse thigh infection model with Gram-positive pathogens (20). It should be noted, however, that the magnitudes of the free-drug AUC_0–24_/MIC ratios associated with bacterial stasis and CFU reductions in the *in vitro* one-compartment E. coli infection system (9) were higher than the ratios associated with efficacy in the present study. These differences likely reflect the environmental disparity between *in vitro* and *in vivo* models (21).

Overall, the results of this study provide strong support for further investigation of gepotidacin as a treatment for UTIs caused by E. coli and are useful in guiding the selection of dosing regimens for clinical trials. Given the critical need to address the rapidly increasing antibiotic resistance of E. coli isolates identified in community and hospital settings (22), gepotidacin may offer the potential to address these infections.

## MATERIALS AND METHODS

### Bacterial isolates and inoculum preparation.

Four *in vivo*-passaged MDR isolates of E. coli were used in this study: IR5, 5649, ALL, and NCTC13441. The E. coli IR5, 5649, and ALL isolates harbor New Delhi metallo-β-lactamase 1. The NCTC13441 isolate is a uropathogenic sequence type 131 levofloxacin-resistant strain. The MICs of gepotidacin and levofloxacin against the four isolates were previously determined (9). The MIC assays were performed in triplicate using standard methodology for broth microdilution, following Clinical and Laboratory Standards Institute guidelines (23, 24); modal values are shown in Table 1.

Bacterial isolates were stored at −80°C and freshly subcultured prior to use. To prepare inocula for the infection of rats, 0.1 ml of thawed stock culture was added to a flask containing 50 ml of brain heart infusion broth (Becton Dickinson, Sparks, MD) and grown overnight at 37°C with gentle agitation (100 rpm). A log-phase culture was then prepared by growing 1 ml of the overnight culture in 50 ml of fresh brain heart infusion broth for 3 h at 37°C with gentle agitation. The log-phase culture was washed once by centrifugation (5 min, 5,000 rpm), and the pellet was reconstituted in sterile saline (0.9% [wt/vol] sodium chloride; Baxter Healthcare Corp., Deerfield, IL). Inocula were prepared by diluting the reconstituted suspension 1:10, 1:50, or 1:500 in sterile saline to achieve approximately 7.0 to 8.4 log_10_ CFU/ml.

### Animals.

Specific-pathogen-free male Sprague-Dawley rats (Charles River, Raleigh, NC) weighing ∼175 g were used in these studies. Food and water were provided *ad libitum* throughout the experiments. Animals were housed in custom caging, using a system designed to support controlled, continuous infusion (14).

Rats were cannulated in the carotid artery and/or jugular vein to enable i.v. infusion of test compounds and monitoring of blood levels of compounds during experiments, as previously described (14). Cannulation surgery was performed 4 to 6 days before initiation of experiments under isoflurane (4%) anesthesia. Subcutaneous flunixin meglumine (Banamine; 1.1 mg/kg) was given to rats before and after surgery to reduce pain and inflammation. Groups of five to six animals were used in efficacy experiments; animals treated with gepotidacin in these experiments were also evaluated for drug exposure in blood samples. Drug concentrations in kidney homogenates (and blood samples) were measured in a separate experiment with three animals per group (per time point).

All procedures were performed in accordance with protocols approved by the GlaxoSmithKline Institutional Animal Care and Use Committee and met or exceeded the standards of the American Association for the Accreditation of Laboratory Animal Care, the U.S. Department of Health and Human Services, and all local and federal animal welfare laws.

### Exclusion criteria.

Rats that did not receive full treatment due to technical issues were excluded from analyses. No other exclusion criteria were applied. Across all experiments, only 3% of animals were excluded in total (the highest exclusion rate per study was 6%); no more than one animal per group was excluded in any experiment.

### Infection of rats to induce pyelonephritis.

Four to six days after cannulation surgery, rats were anesthetized by combined intramuscular injection of 100 μl of ketamine hydrochloride (40 mg/kg; Ketaset) and xylazine (5 mg/kg; Rompun). Kidneys were located by external palpation, and a 500-μl inoculum of the log-phase bacterial suspension in saline was injected through the skin into each kidney. Each rat had both kidneys injected (total inoculum = 1 ml/rat, delivering 7.0 to 8.4 log_10_ CFU/rat).

### Administration of antimicrobial test agents.

Gepotidacin and levofloxacin formulations were freshly prepared on each day of administration to rats. Gepotidacin was prepared from a powder (batch 2140944E-C-06P; synthesized at GlaxoSmithKline Pharmaceuticals, Collegeville, PA) as 1- or 3-mg/ml solutions in sterile saline. Levofloxacin was prepared from a commercially available liquid i.v. formulation (lot 051114; Akorn, Inc., Lake Forest, IL) by dilution to 2.5 mg/ml with sterile saline (see Table S1 in the supplemental material).

Starting at 2 h postinfection, the prepared gepotidacin and levofloxacin formulations were administered via the implanted cannulae into the jugular vein of the rats as continuous infusions. Infusion pumps (pump 22; Harvard Instruments, Edenbridge, Kent, UK; with one pump being used per compound and dosing regimen) were programmed to deliver changing flow rates over time to target recreated (in rat blood) plasma concentrations measured in humans (Table S2). For gepotidacin, flow rates were reset q12h to simulate BID dosing. For levofloxacin, flow rates were reset q24h to simulate once-daily dosing. Vehicle control animals received saline at a constant flow rate of 0.4 ml/h.

Gepotidacin was administered to recreate exposure profiles observed in healthy humans on day 16 of q12h oral dosing (800 or 1,500 mg) (8). Levofloxacin was administered q24h to recreate the exposure profile observed in humans given a single oral dose of 500 mg (25). Plasma protein binding for gepotidacin was similar between rat and human (34 and 33%, respectively) (data on file). Levofloxacin protein binding was also similar between the species (26, 27). Therefore, target exposure profiles in rat and human were similar based on both total and free-drug concentrations.

For efficacy experiments, dosing continued for 4 days (eight doses [q12h] total for gepotidacin and four doses [q24h] total for levofloxacin). For assessment of gepotidacin concentrations in rat blood and kidneys, three total doses using a q12h regimen were administered with samples taken at various times after the third dose.

### Determination of gepotidacin and levofloxacin exposures in rat blood.

Exposure profiles for gepotidacin in rat blood were established in preliminary PK studies using infected rats (data on file) and confirmed in each of the efficacy studies. Blood (∼100 μl) was collected from infected rats at 0, 0.5, 1, 1.5, 2, 3, 4, 6, 8, and/or 12 h after the start of infusion of the third dose of gepotidacin in a q12h regimen (*n* = 5 to 6 rats per time point). Blood was collected via implanted cannulae from the carotid artery into EDTA-coated tubes. A 25-μl aliquot of each sample was mixed with 25 μl of high-pressure liquid chromatography (HPLC)-grade water and immediately frozen on dry ice. All samples were stored at −80°C until analysis using HPLC-tandem mass spectroscopy (MS/MS) with electrospray ionization working in multiple-reaction-monitoring mode with a Waters Acquity ultrahigh-performance liquid chromatograph connected to an API Sciex 4000 tandem quadrupole mass spectrometer (performed at GlaxoSmithKline, Collegeville, PA). The LLQ for gepotidacin in rat blood was 0.05 μg/ml.

Exposure profiles for levofloxacin in rat blood were determined in preliminary PK studies using uninfected rats and subsequently confirmed in historical experiments with infected rats using methods similar to those described above (data on file) The LLQ for levofloxacin in rat blood was 0.01 μg/ml.

### Measurement of gepotidacin concentrations in rat kidneys.

A separate experiment was performed to determine concentrations of gepotidacin in rat kidneys. Infected rats were euthanized at 0, 2, 4, 6, 8, and 12 h after the start of infusion of the third dose of gepotidacin in a q12h regimen for the 1,500-mg recreated dose (*n* = 3 rats per time point). Blood (∼500 μl) was collected via cardiac puncture and transferred immediately into EDTA-coated tubes. Blood samples were processed, stored, and analyzed as described above. Kidneys were removed, weighed, and processed individually. Each kidney was homogenized in 9 ml of acetonitrile using a laboratory blender (Stomacher 80; Seward, Ltd., Worthing, UK). Homogenates were centrifuged at 4°C at 1,800 × *g* for 10 min. A 50-μl aliquot of the supernatant was removed and maintained at 5°C until analysis by HPLC-MS/MS (performed at GlaxoSmithKline). The LLQ for gepotidacin in rat kidney homogenate was 0.025 μg/g tissue. Measured concentrations were adjusted with a correction factor based on kidney weight (in grams), and a volume of acetonitrile (9 ml) was added for homogenization ([weight + 9]/weight). All final concentrations were considered to come from independent samples for mathematical calculations (e.g., six samples at each time point for left and right kidneys from *n* = 3 rats).

### Analysis of antibacterial efficacy.

Efficacy was evaluated by determining bacterial counts in homogenates of kidneys (primary endpoint) and bladders (secondary endpoint) collected from rats euthanized by carbon dioxide overdose at the following times postinfection (*n* = 5 to 6 rats per time point): (i) 2 and 96 h (4-day treatment experiment; 800- and 1,500-mg q12h dosing) or (ii) 2, 24, 48, 72, and 96 h (time course experiment; 1,500-mg q12h dosing only).

For the 2-h time point, rats were euthanized just before treatment initiation, thus providing nontreated baseline control samples. The 96-h time point corresponded to the end of the final infusion of test article (12 or 24 h after start of the final infusion).

Kidneys and bladders were excised aseptically from euthanized animals and homogenized in 1 ml/organ sterile saline using a laboratory blender. Both kidneys from each animal were combined for homogenization and bacterial enumeration. Tenfold serial dilutions of homogenates were prepared in saline using a Hamilton AT2Plus liquid handling system and inoculated (20 μl/plate) in triplicate onto Trypticase soy agar plates supplemented with 5% sheep’s blood. Colonies were counted after overnight incubation at 37°C. The limit of detection was 1.2 log_10_ CFU/tissue. Total drug concentrations of gepotidacin measured in the kidney homogenates were close to or below the broth MIC by 12 h after the start of infusion, and there was no evidence of *ex vivo* bacterial killing on the plates (data not shown).

Systemic drug exposure was also evaluated in 4-day treatment efficacy experiments using blood samples collected as described above.

### Data handling and analysis.

Efficacy data (log_10_ CFU/tissue) are presented as per-group means ± the standard deviations (SD). Statistical comparisons between treatment groups were performed using Student *t* test, with *P* values of ≤0.05 considered significant.

Exposure data in rat blood and kidneys are presented as mean concentrations ± the SD in μg/ml or μg/g, respectively. The concentrations of gepotidacin in kidney homogenates were adjusted to account for their dilution with acetonitrile. Blood and tissue densities were assumed to be 1 g/ml per standard drug metabolism and PK procedure, allowing kidney results in μg/g to be directly compared to blood results in μg/ml. To compare blood and kidney concentrations from the same animals, a pseudoprofile bootstrap resampling approach was used to create composite exposure profiles whereby the first sample taken at each time point was assigned to profile 1, the second to profile 2, etc.

The *C*_max_ was determined to be the highest concentration achieved in the averaged profiles. The AUC for a single dose was calculated from the averaged profiles using the trapezoid rule from time zero to the last measured time point (12 h for gepotidacin and 24 h for levofloxacin). Daily gepotidacin AUC values were determined by multiplying the single dose value by 2 for the q12h dosing regimen.

### Data availability.

On reasonable request, materials and data will be made available in a timely fashion, at reasonable cost, and in limited quantities to members of the scientific community for noncommercial purposes.

## Supplementary Material

Supplemental file 1
