# Supplementary material for: Efficacy of Human Exposures of Gepotidacin (GSK2140944) against Escherichia coli in a Rat Pyelonephritis Model
Source: Antimicrob Agents Chemother. 2019 Jun 24;63(7):e00086-19. doi: 10.1128/AAC.00086-19 (PMC6591613; doi:10.1128/AAC.00086-19)
Supplement: Supplemental file 1 [file AAC.00086-19-s0001.pdf]

SUPPLEMENTAL MATERIAL

Efficacy of Human Exposures of Gepotidacin (GSK2140944) Against *Escherichia coli* in a Rat Pyelonephritis Model

Methods

Delivery of Infusions:

Flow rates for each treatment were pre-determined in PK studies prior to conducting the efficacy experiments. Note that flow rates depend on multiple factors including (1) syringe size and make; (2) infusion tubing diameter; and (3) concentration of infusion solution. In these studies, all tubing and catheters were made of polyethylene and had an outer diameter of 0.8 mm and inner diameter of 0.4 mm. The concentrations of the solutions for infusion are shown in Supplementary Table 1. Under this set of conditions, the flow rates as shown in Supplementary Table 2 produced the exposure profiles provided in the manuscript figures. The infusion pumps started with an initial flow rate as indicated by time 0; at varying time intervals thereafter, the rate changed to the corresponding value listed in the table. Note that the flow rates for gepotidacin were modified slightly after the first two experiments to more closely reproduce the human profiles.

Table S1. Concentrations of each treatment solution prepared for infusion

Treatment	Concentration of infused solution
Levofloxacin 500 mg QD	2.5 mg/mL
Gepotidacin 800 mg BID	1 mg/mL
Gepotidacin 1500 mg BID	3 mg/mL
BID, administered every 12 hours; QD, administered every 24 hours.	

Table S2. Flow rates delivered over the dosing interval for each treatment

Time (hour)	Flow Rate (mL/hour)		
	Levofloxacin 500 mg QD	Gepotidacin 800 mg BID	Gepotidacin 1500 mg BID
0	1.58	0.25[a], 0.3[b]	0.25
0.25	1.39	0.25[a], 0.3[b]	0.25
0.5	1.24	0.25[a], 0.3[b]	0.25
0.75	1.12	0.25[a], 0.3[b]	0.25
1	1.02	0.9[a], 1.0[b]	1.0
1.25	0.94	0.9[a], 1.0[b]	1.0
1.5	0.87	0.9[a], 1.0[b]	1.0
1.75	0.81	0.9[a], 1.0[b]	0.5[a], 1.0[b]
2	0.77	0.6	0.25[a], 0.3[b]
2.25	0.73	0.6	0.25[a], 0.3[b]
2.5	0.69	0.6	0.25[a], 0.3[b]
2.75	0.66	0.6	0.25[a], 0.3[b]
3	0.65	0.4	0.2[a], 0.25[b]
3.25	0.63	0.4	0.2[a], 0.25[b]
3.5	0.62	0.4	0.2[a], 0.25[b]

Time (hour)	Flow Rate (mL/hour)		
	Levofloxacin 500 mg QD	Gepotidacin 800 mg BID	Gepotidacin 1500 mg BID
3.75	0.6	0.4	0.2[a], 0.25[b]
4	0.59	0.3	0.2
4.25	0.58	0.23	0.17
4.5	0.56	0.17	0.14
4.75	0.55	0.1	0.1
5	0.53	0.1	0.1
5.25	0.52	0.1	0.1
5.5	0.51	0.1	0.1
5.75	0.49	0.1	0.1
6	0.48	0.09	0.09
6.25	0.46	0.09	0.09
6.5	0.45	0.09	0.09
6.75	0.44	0.09	0.09
7	0.42	0.08	0.08
7.25	0.41	0.08	0.08
7.5	0.39	0.08	0.08
7.75	0.37	0.08	0.08
8	0.36	0.07	0.07
8.25	0.36	0.07	0.07
8.5	0.35	0.07	0.07
8.75	0.34	0.07	0.07
9	0.34	0.06	0.06
9.25	0.34	0.06	0.06
9.5	0.33	0.06	0.06
9.75	0.32	0.06	0.06
10	0.32	0.05	0.05
10.25	0.31	0.05	0.05

Time (hour)	Flow Rate (mL/hour)		
	**Levofloxacin 500 mg QD**	**Gepotidacin 800 mg BID**	**Gepotidacin 1500 mg BID**
10.5	0.31	0.05	0.05
10.75	0.3	0.05	0.05
11	0.29	0.04	0.04
11.25	0.29	0.04	0.04
11.5	0.28	0.04	0.04
11.75	0.28	0.03	0.03
12	0.27	Repeat starting at 0 hour rate	Repeat starting at 0 hour rate
12.25	0.26		
12.5	0.26		
12.75	0.25		
13	0.25		
13.25	0.24		
13.5	0.23		
13.75	0.21		
14	0.2		
16	0.16		
18	0.13		
20	0.11		
22	0.09		
24	Repeat starting at 0 hour rate	Repeat starting at 0 hour rate	Repeat starting at 0 hour rate

[a] Flow rate used against *E. coli* IR5 and ALL.
[b] Flow rate used against *E. coli* 5649 and NCTC13441.
BID, administered every 12 hours; QD, administered every 24 hours.

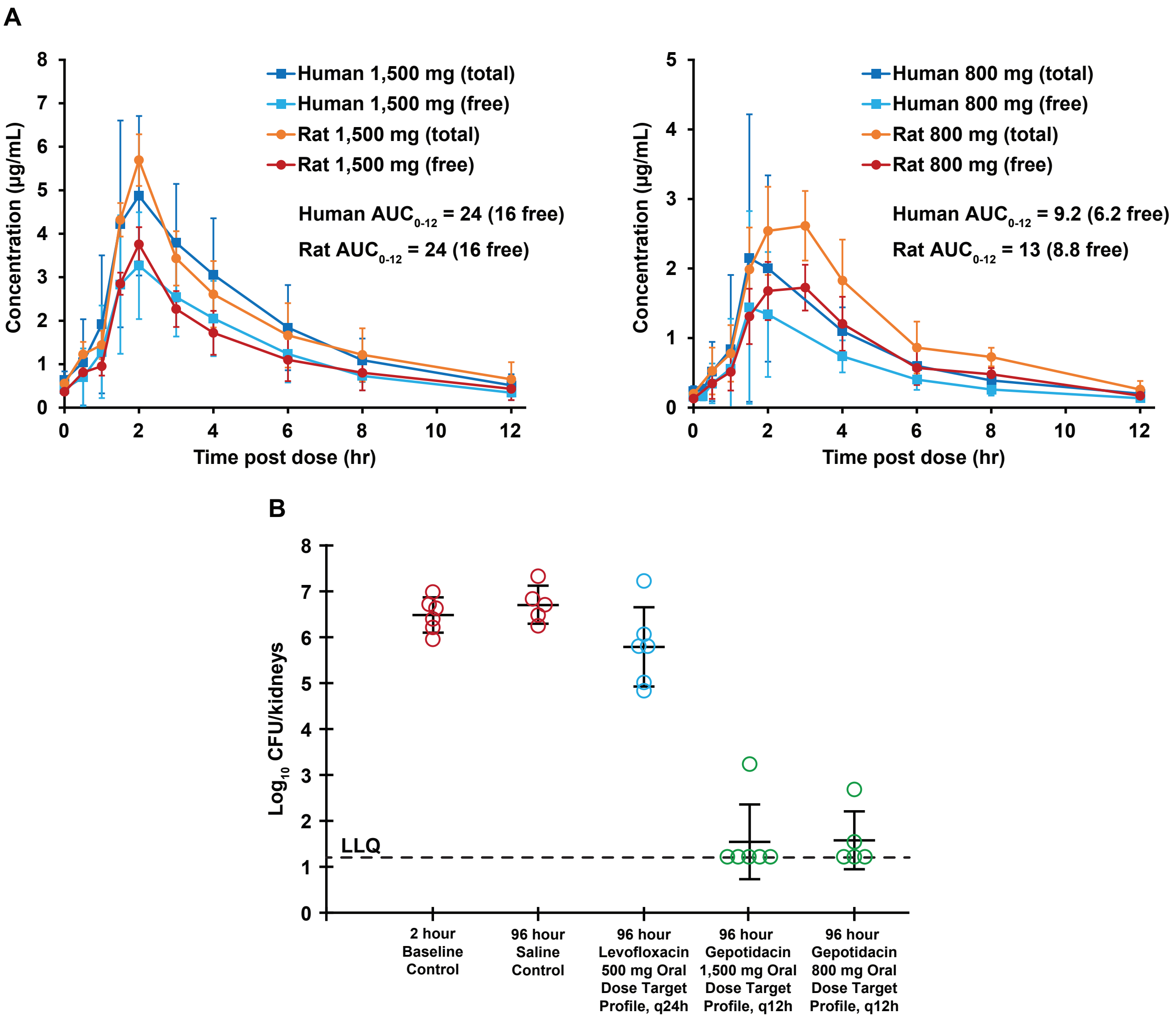

FIG S1. Exposure and efficacy in rats infected with *E. coli* NCTC13441 isolate. (A) Total and free-drug mean systemic exposure profiles for human target concentrations and those measured in rats during the *E. coli* NCTC13441 isolate study. (B) Efficacy against *E. coli* NCTC13441 isolate (uropathogenic sequence type 131 [ST-131] gepotidacin MIC 4 µg/mL). N = 5 to 6 rats/time point; circles show data for individual animals, horizontal lines show the group mean flanked by the standard deviation. hr, hours; LLQ, lower limit of quantification.

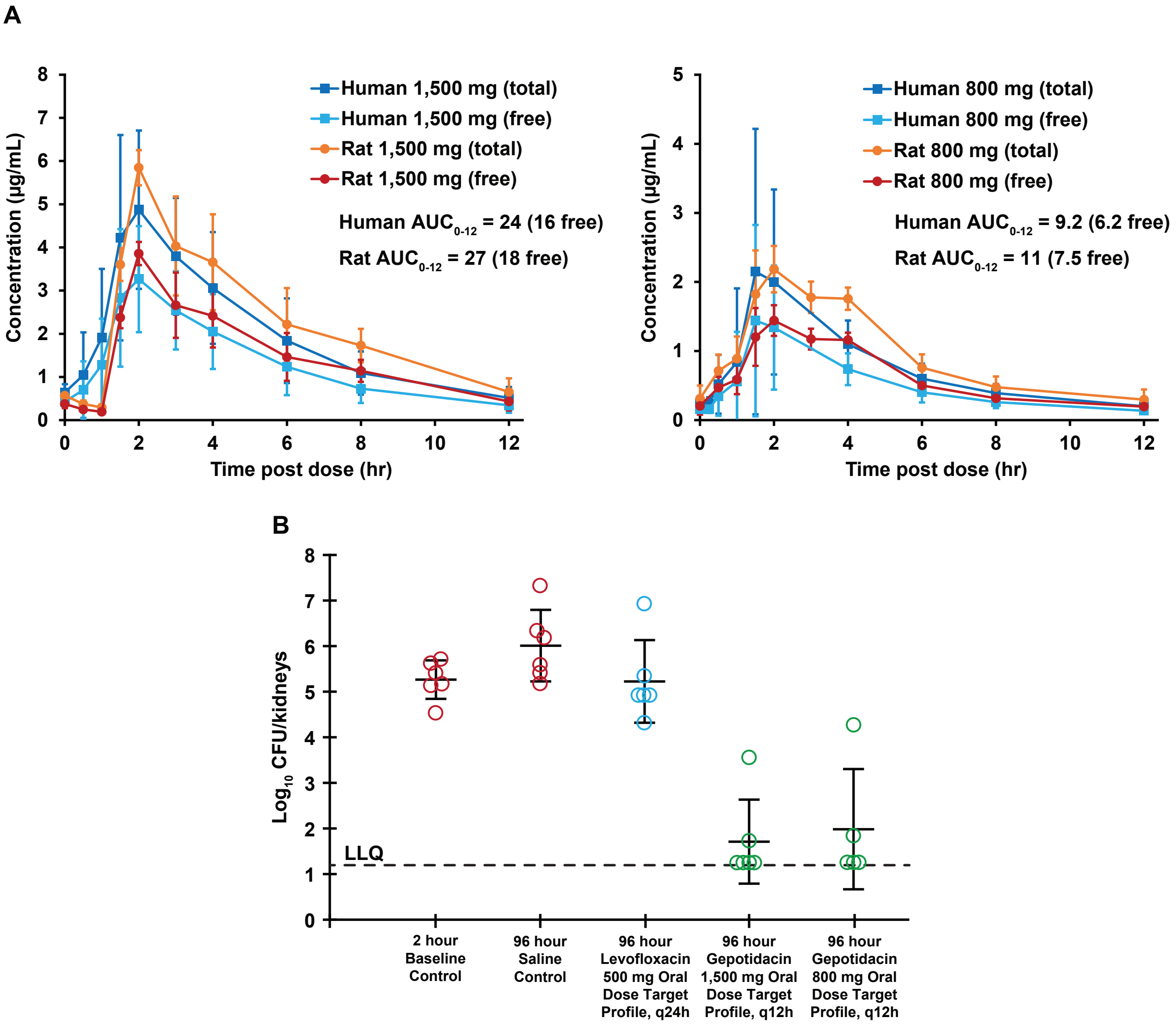

FIG S2. Exposure and efficacy in rats infected with *E. coli* 5649 isolate. (A) Total and free-drug mean systemic exposure profiles for human target concentrations and those measured in rats during the *E. coli* 5649 isolate study. (B) Efficacy against *E. coli* 5649 isolate (NDM-1; gepotidacin MIC 2 µg/mL). N = 5 to 6 rats/time point; circles show data for individual animals, horizontal lines show the group mean flanked by the standard deviation. hr, hours; LLQ, lower limit of quantification.

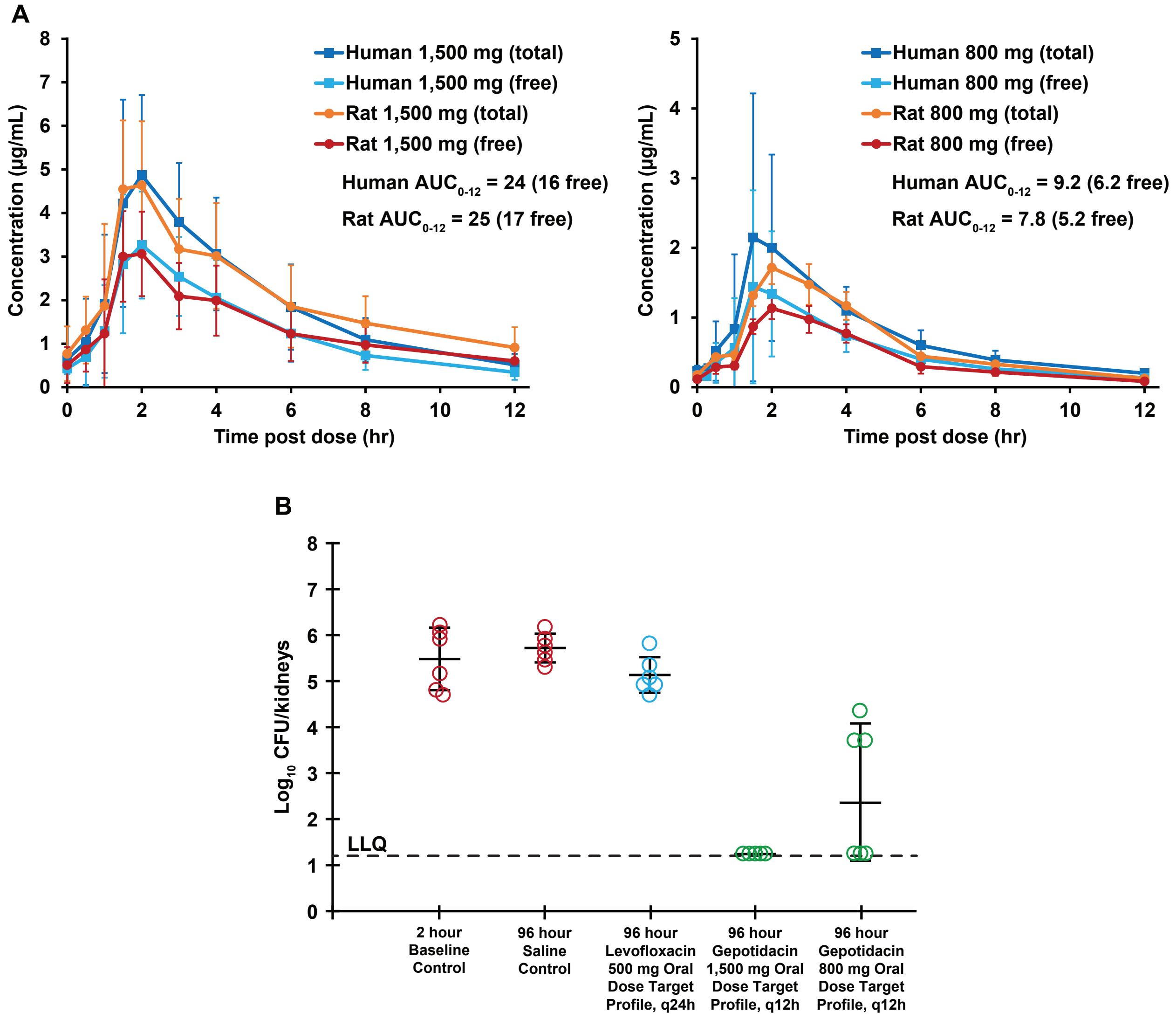

FIG S3. Exposure and efficacy in rats infected with *E. coli* IR5 isolate. (A) Total and free-drug mean systemic exposure profiles for human target concentrations and those measured in rats during the *E. coli* IR5 isolate study. (B) Efficacy against *E. coli* IR5 isolate (NDM-1; gepotidacin MIC 4 µg/mL). N = 5 to 6 rats/time point; circles show data for individual animals, horizontal lines show the group mean flanked by the standard deviation. hr, hours; LLQ, lower limit of quantification.

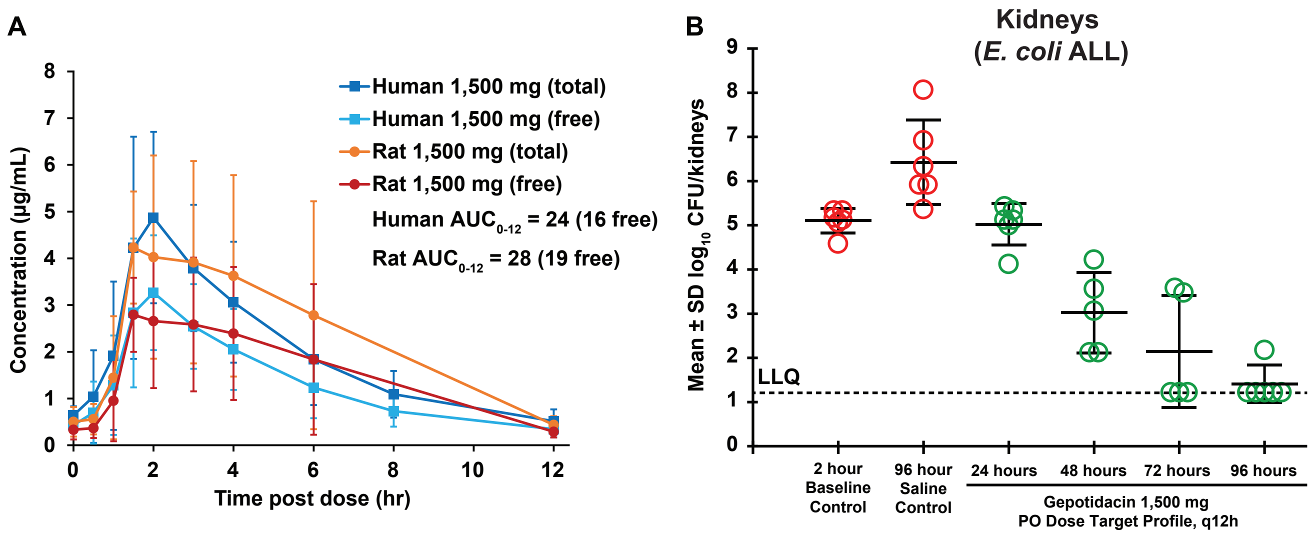

FIG S4. Exposure and efficacy in rats infected with *E. coli* ALL isolate. (A) Total and free-drug mean systemic exposure profiles for 1,500-mg human target concentrations and those measured in rats during the *E. coli* ALL time-course study. (B) Efficacy against *E. coli* ALL isolate (NDM-1; gepotidacin MIC 4 µg/mL). N = 5 to 6 rats/ time point; circles show data for individual animals, horizontal lines show the group mean flanked by the standard deviation. hr, hours; LLQ, lower limit of quantification.

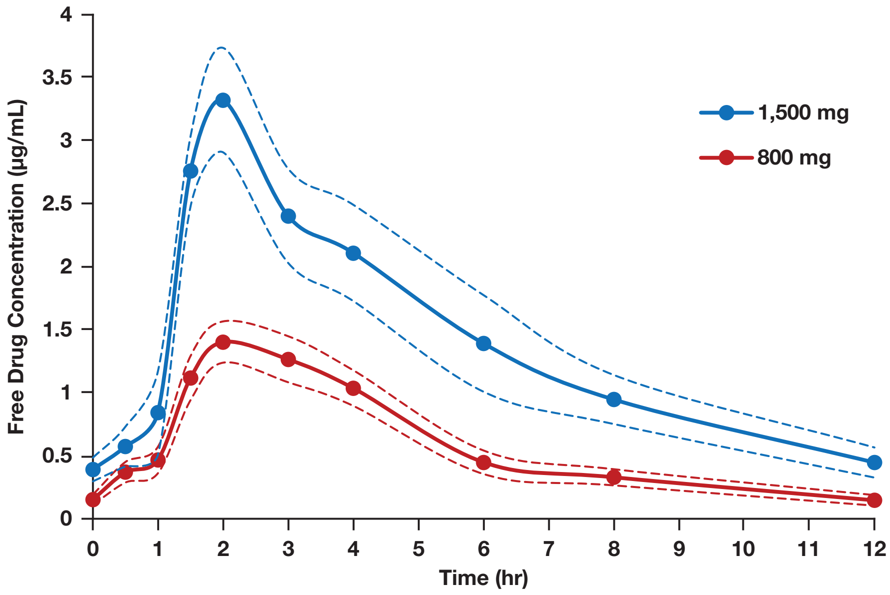

FIG S5. Pooled mean blood free-drug gepotidacin exposure profiles achieved in rats across all experiments, shown with 95% confidence intervals (dashed lines). Daily free-drug AUC_{0-12} values for the 95% lower bound, the mean profile, and the 95% upper bound were 27, 34, and 40 µg•h/mL for the 1,500-mg exposure profile and 12, 14, and 16 µg•h/mL for the 800-mg exposure profile. hr, hours.
